# Supplementary material for: Radiocarbon constraints on the glacial ocean circulation and its impact on atmospheric CO2
Source: Nat Commun. 2017 Jul 13;8:16010. doi: 10.1038/ncomms16010 (PMC5511348; doi:10.1038/ncomms16010)
Supplement: Supplementary Information [file ncomms16010-s1.pdf]

File name: Supplementary Information
Description: Supplementary Figures, Supplementary Tables and Supplementary References

File name: Peer Review File
Description:

File name: Supplementary Data 1
Description: New and compiled foraminifer and coral radiocarbon data referred to in this study. Full references for compiled data are provided in Supplementary Table 2. Modern radiocarbon ventilation ages (and surface reservoir ages) are interpolated from the GLODAP data base (Key et al., 2004).

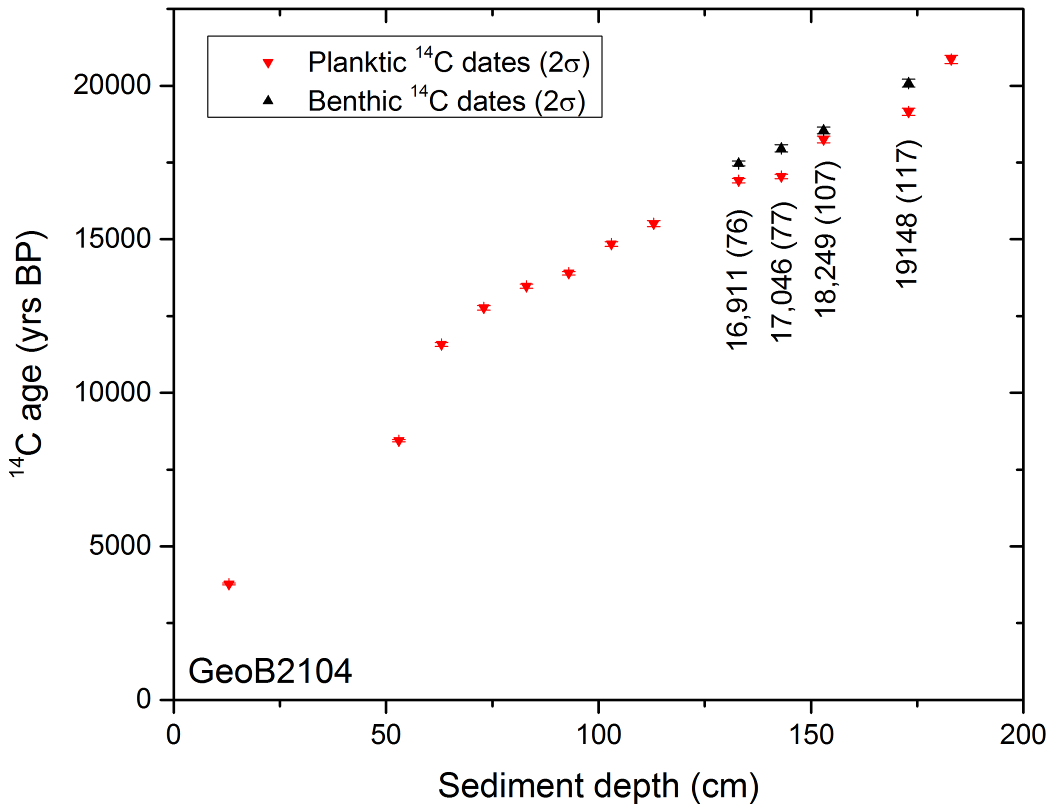

Supplementary Figure 1. Deglacial chronostratigraphy for core GeoB2014 (-27.3°N, 46.4°W,1503m), illustrated by uncorrected planktonic (and LGM benthic) radiocarbon dates, with 2 sigma errors. Numbers indicate uncorrected planktonic radiocarbon ages for the LGM samples included in this compilation (with 2 sigma errors).

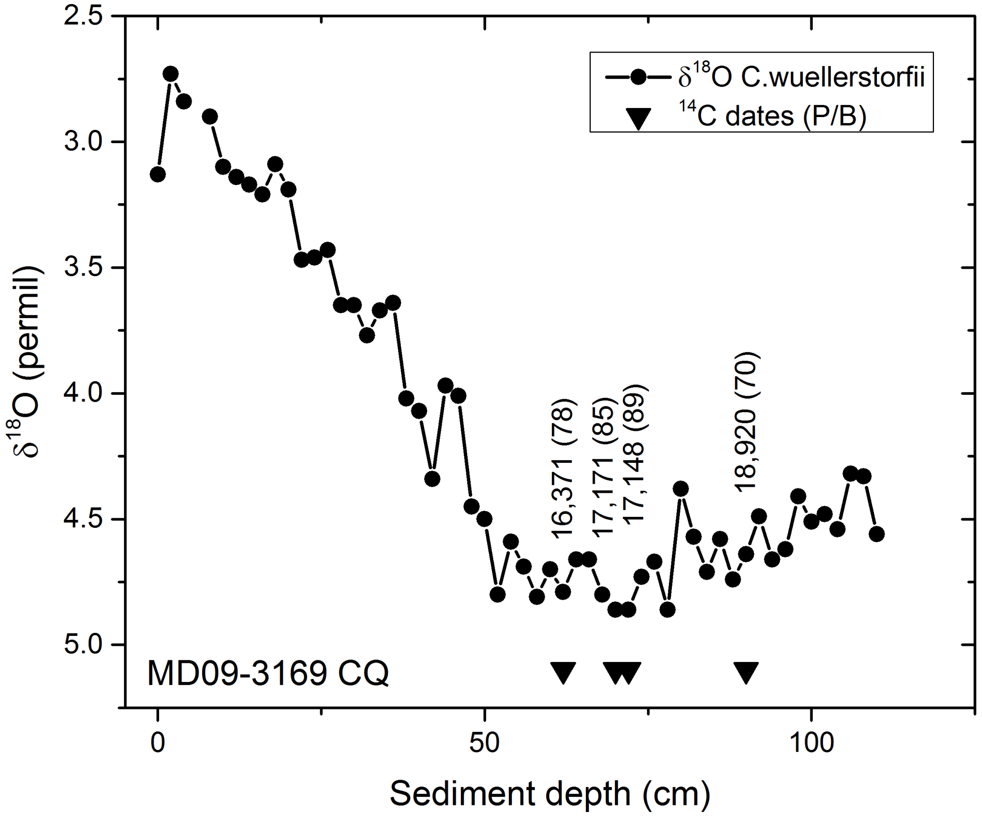

Supplementary Figure 2. Deglacial benthic isotope stratigraphy for core MD09-3169CQ (-23.4^{o}N, 11.7^{o}W, 2975m). Uncorrected planktonic radiocarbon dates (2 sigma errors) and their locations in the core are indicated by the solid triangles.

Supplementary Table 1. Sediment cores used for new data presented in this study

Core	Strat. Reference	Basin	Lat (N)	Long(W)	Depth
TR163-31B	Shackleton et al. (1989) [1]	EEP	-3.0	84.0	3210
TR163-22	Lea et al. (2006) [2]	EEP	0.5	92.0	2830
SK129-CR2	Piotrowski et al. (2009) [3]	I	3.0	-76.0	3800
ODP-1240	de la Fuente et al. (2015) [4]	EEP	0.0	86.5	2921
MD98-2165	Waelbroeck et al. (2006) [5]	I	-9.6	-118.3	2100
MD97-2121	Skinner et al. (2015) [6]	SWP	-40.4	-178.0	2314
CHAT-5K	McCave et al. (2008) [7]	SWP	-40.8	171.5	4240
CHAT-3K	McCave et al. (2008) [7]	SWP	-42.7	167.5	4802
CHAT-16K	McCave et al. (2008) [7]	SWP	-42.5	178.5	1408
CHAT-10K	McCave et al. (2008) [7]	SWP	-40.0	180.0	3003
CD38-17	Sadekov et al. (2013) [8]	EEP	-1.4	90.3	2580
MD08-3169	(this study, Suppl. Fig. 3)	SEA	-23.4	11.7	2975
GeoB-2104	(this study, Suppl. Fig. 2)	SWA	-27.3	-46.4	1503

Supplementary Table 2. Locations and references corresponding to site locations given in Supplementary Data 1 and Figure 1 of the main text.

CORE	Reference.	Site number	Lat (N)	Long (W)	Water Depth
MD97-2121	Skinner et al. (2015) [6]	1	-40.4	-178.0	2314
MD97-2121	this study	1	-40.4	-178.0	2314
MD97-2121	this study	1	-40.4	-178.0	2314
MD97-2121	this study	1	-40.4	-178.0	2314
MD97-2121	this study	1	-40.4	-178.0	2314
MD97-2121	Skinner et al., (2015) [6]	1	-40.4	-178.0	2314
SO213-82-1	Ronge et al. (2016) [9]	2	-45.8	-176.6	2066
SO213-84-1	Ronge et al. (2016) [9]	3	-45.1	-174.6	972
Nesmeyanov GGC-20	Keigwin & Schlegel (2002) [10]	4	48.9	-150.4	1510
Nesmeyanov GGC-18	Keigwin & Schlegel (2002) [10]	5	48.9	-150.4	1700
Nesmeyanov GGC-15	Keigwin & Schlegel (2002) [10]	6	48.9	-150.4	1980
B34-91	Keigwin & Schlegel (2002) [10]	7	49.1	-150.3	1227
Nesmeyanov GGC-27	Keigwin & Schlegel (2002) [10]	8	49.6	-150.2	995
SH_SOI_A04	Hines et al. (2016) [11]	9	-45.0	-148.0	1575
SH_SOI_F06	Hines et al. (2016) [11]	10	-45.0	-148.0	1523
SH_SOI_E03	Hines et al. (2016) [11]	11	-45.0	-148.0	1460
SH_SOI_G10-2	Hines et al. (2016) [11]	12	-45.0	-148.0	1575
SH_SOI_E04	Hines et al. (2016) [11]	13	-45.0	-148.0	1460
SH_SOI_F05	Hines et al. (2016) [11]	14	-45.0	-148.0	1523
SH_SOI_F04-2	Hines et al. (2016) [11]	15	-45.0	-148.0	1523
SH_SOI_E02	Hines et al. (2016) [11]	16	-45.0	-148.0	1460
MD97-2138	Broecker & Clark (2011) [12]	17	-1.0	-146.0	1900
MR01-K03 PC4/PC5	Ahagon et al. (2003) [13]	18	41.1	-142.4	1366
MR01-K03 PC4/PC5	Ahagon et al. (2003) [13]	18	41.1	-142.4	1366
MD01-2420	Okazaki et al. (2012) [14]	19	36.1	-141.8	2101
MD01-2420	Okazaki et al. (2012) [14]	19	36.1	-141.8	2101
MD01-2420	Okazaki et al. (2012) [14]	19	36.1	-141.8	2101
KT89-18-P4	Murayama et al. (1992) [15]	20	-32.2	-133.9	2700
KT89-18-P4	Murayama et al. (1992) [15]	20	-32.2	-133.9	2700
MD01-2386	Broecker at al. (2008) [16]	21	1.1	-129.8	2820
MD01-2386	Broecker at al. (2008) [16]	21	1.1	-129.8	2820
MD01-2386	Broecker at al. (2008) [16]	21	1.1	-129.8	2820
MD98-2181	Broecker et al. (2004) [17]	22	6.0	-126.0	2100
MD98-2181	Broecker et al. (2004) [17]	22	6.0	-126.0	2100
MD98-2181	Broecker et al. (2004) [17]	22	6.0	-126.0	2100
MD98-2165	this study	23	-9.6	-118.3	2100
MD98-2165	this study	23	-9.6	-118.3	2100
MD98-2165	this study	23	-9.6	-118.3	2100
MD98-2165	this study	23	-9.6	-118.3	2100
RC27-14	Bryan et al. (2010) [18]	24	18.3	-57.7	596
RC27-14	Bryan et al. (2010) [18]	24	18.3	-57.7	596

RC27-23	Bryan et al. (2010) [18]	25	18.0	-57.6	820
RC27-23	Bryan et al. (2010) [18]	25	18.0	-57.6	820
RC27-23	Bryan et al. (2010) [18]	25	18.0	-57.6	820
RC27-23	Bryan et al. (2010) [18]	25	18.0	-57.6	820
RC27-23	Bryan et al. (2010) [18]	25	18.0	-57.6	820
RC27-23	Bryan et al. (2010) [18]	25	18.0	-57.6	820
TNO57-21	Barker et al. (2010) [19]	26	-41.1	-7.8	4981
TNO57-21	Barker et al. (2010) [19]	26	-41.1	-7.8	4981
TNO57-21	Barker et al. (2010) [19]	26	-41.1	-7.8	4981
TNO57-21	Barker et al. (2010) [19]	26	-41.1	-7.8	4981
TNO57-21	Barker et al. (2010) [19]	26	-41.1	-7.8	4981
PS-1243-1	Thornalley et al. (2015) [20]	27	69.4	-6.6	2711
PS-1243-1	Thornalley et al. (2015) [20]	27	69.4	-6.6	2711
PS-1243-1	Thornalley et al. (2015) [20]	27	69.4	-6.6	2711
SHAK10-10K	Freeman et al. (2016) [21]	28	37.8	9.5	1127
SHAK10-10K	Freeman et al. (2016) [21]	28	37.8	9.5	1127
SHAK14-4G	Freeman et al. (2016) [21]	29	37.8	9.7	2063
SHAK14-4G	Freeman et al. (2016) [21]	29	37.8	9.7	2063
SHAK05-3K	Freeman et al. (2016) [21]	30	37.6	9.7	4670
SHAK05-3K	Freeman et al. (2016) [21]	30	37.6	9.7	4670
SHAK05-3K	Freeman et al. (2016) [21]	30	37.6	9.7	4670
MD99-2334K	Skinner et al. (2014) [22]	31	38.0	10.0	3146
MD99-2334K	Skinner et al. (2014) [22]	31	38.0	10.0	3146
MD99-2334K	Skinner et al. (2014) [22]	31	38.0	10.0	3146
SHAK03-6K	Freeman et al. (2016) [21]	32	37.7	10.5	3735
SHAK03-6K	Freeman et al. (2016) [21]	32	37.7	10.5	3735
SHAK03-6K	Freeman et al. (2016) [21]	32	37.7	10.5	3735
SHAK03-6K	Freeman et al. (2016) [21]	32	37.7	10.5	3735
MD08-3169	this study	33	-23.4	11.7	2975
MD08-3169	this study	33	-23.4	11.7	2975
MD08-3169	this study	33	-23.4	11.7	2975
MD08-3169	this study	33	-23.4	11.7	2975
RC24-08	Cleroux et al. (2011) [23]	34	-1.3	11.9	550
MD07-3076	Skinner et al. (2010) [24]	35	-44.1	14.2	3770
MD07-3076	Skinner et al. (2010) [24]	35	-44.1	14.2	3770
MD07-3076	Skinner et al. (2010) [24]	35	-44.1	14.2	3770
MD07-3076	Skinner et al. (2010) [24]	35	-44.1	14.2	3770
MD07-3076	Skinner et al. (2010) [24]	35	-44.1	14.2	3770
RAPID-17-5P	Thornalley et al. (2011) [25]	36	61.0	19.0	2303
RAPID-17-5P	Thornalley et al. (2011) [25]	36	61.0	19.0	2303
RAPID-17-5P	Thornalley et al. (2011) [25]	36	61.0	19.0	2303
f0076carcs011	Chen et al. (2015) [26]	37	5.6	27.0	845
MD09-3256	Freeman et al. (2016) [21]	38	-3.5	35.4	3537
MD09-3256	Freeman et al. (2016) [21]	38	-3.5	35.4	3537
MD09-3256	Freeman et al. (2016) [21]	38	-3.5	35.4	3537
MD09-3256	Freeman et al. (2016) [21]	38	-3.5	35.4	3537
MD09-3257	Freeman et al. (2016) [21]	39	-4.3	36.3	2344

MD09-3257	Freeman et al. (2016) [21]	39	-4.2	36.4	2344
GS07-150-17/1A	Freeman et al. (2016) [21]	40	-4.2	37.1	1000
GS07-150-17/1A	Freeman et al. (2016) [21]	40	-4.2	37.1	1000
GS07-150-17/1A	Freeman et al. (2016) [21]	40	-4.2	37.1	1000
GS07-150-17/1A	Freeman et al. (2016) [21]	40	-4.2	37.1	1000
f0115solnm002d	Chen et al. (2015) [26]	41	10.7	44.6	1097
f0115solnm002c	Chen et al. (2015) [26]	42	10.7	44.6	1097
f0115solnm002f	Chen et al. (2015) [26]	43	10.7	44.6	1097
f0115solnm002a	Chen et al. (2015) [26]	44	10.7	44.6	1097
GeoB-2104	this study	45	-27.3	46.4	1503
GeoB-2104	this study	45	-27.3	46.4	1503
GeoB-2104	this study	45	-27.3	46.4	1503
GeoB-2104	this study	45	-27.3	46.4	1503
KNR-159-5-36GGC	Sortor and Lund (2011) [27]	46	-27.5	46.5	1268
KNR-159-5-36GGC	Sortor and Lund (2011) [27]	46	-27.5	46.5	1268
KNR-159-5-36GGC	Sortor and Lund (2011) [27]	46	-27.5	46.5	1268
KNR-159-5-36GGC	Sortor and Lund (2011) [27]	46	-27.5	46.5	1268
KNR-159-5-36GGC	Sortor and Lund (2011) [27]	46	-27.5	46.5	1268
f0186carcs005	Chen et al. (2015) [26]	47	14.9	48.2	795
NBP1103-DH40-Dc-3	Chen et al. (2015) [26]	48	-60.2	57.8	806
NBP1103-DH43-Dc-6	Chen et al. (2015) [26]	49	-60.2	57.8	823
NBP1103-DH43-Dc-1	Chen et al. (2015) [26]	50	-60.2	57.8	823
NBP1103-DH40-Dc-5	Chen et al. (2015) [26]	51	-60.2	57.8	806
NBP0805_DR23_Dc-A-4	Burke & Robinson (2012) [28]	52	-57.8	64.0	819
NBP0805_DR27_Dc-A-1	Burke & Robinson (2012) [28]	53	-57.8	64.0	1134
SO161-SL22	De Pol Holz et al. (2010) [29]	54	-36.2	73.7	1000
SO161-SL22	De Pol Holz et al. (2010) [29]	54	-36.2	73.7	1000
SO161-SL22	De Pol Holz et al. (2010) [29]	54	-36.2	73.7	1000
SO161-SL22	De Pol Holz et al. (2010) [29]	54	-36.2	73.7	1000
SO161-SL22	De Pol Holz et al. (2010) [29]	54	-36.2	73.7	1000
SO161-SL22	De Pol Holz et al. (2010) [29]	54	-36.2	73.7	1000
SO161-SL22	De Pol Holz et al. (2010) [29]	54	-36.2	73.7	1000
SO161-SL22	De Pol Holz et al. (2010) [29]	54	-36.2	73.7	1000
SO161-SL22	De Pol Holz et al. (2010) [29]	54	-36.2	73.7	1000
MD07-3088	Siani et al. (2013) [30]	55	-46.0	75.0	1536
MD07-3088	Siani et al. (2013) [30]	55	-46.0	75.0	1536
MD07-3088	Siani et al. (2013) [30]	55	-46.0	75.0	1536
MD07-3088	Siani et al. (2013) [30]	55	-46.0	75.0	1536
MD07-3088	Siani et al. (2013) [30]	55	-46.0	75.0	1536
KNR140 37 JPC	Keigwin and Schlegel (2002) [10]	56	31.7	75.4	2972
KNR140 39 GGC	Keigwin and Schlegel (2002) [10]	57	31.7	75.4	2975
TRI 163-31B	Shackleton et al. (1988) [1]	58	-3.6	84.0	3210
TRI 163-31B	this study	58	-3.0	84.0	3210
TRI 163-31B	this study	58	-3.0	84.0	3210
ODP-1240	this study	59	0.0	86.5	2921
ODP-1240	de la Fuente et al. (2015) [4]	59	0.0	86.5	2921
ODP-1240	de la Fuente et al. (2015) [4]	59	0.0	86.5	2921

ME0005-24JC	Keigwin & Lehman (2015) [31]	60	0.0	86.5	2941
VM21-30	Stott et al. (2009) [32]	61	-1.1	89.7	617
VM21-30	Stott et al. (2009) [32]	61	-1.1	89.7	617
VM21-30	Stott et al. (2009) [32]	61	-1.1	89.7	617
VM21-30	Stott et al. (2009) [32]	61	-1.1	89.7	617
CD38-17	this study	62	-1.4	90.3	2580
CD38-17	this study	62	-1.4	90.3	2580
TR163-22	this study	63	0.5	92.0	2830
TR163-22	this study	63	0.5	92.0	2830
TR163-22	this study	63	0.5	92.0	2830
PLDS 7G	Keigwin & Lehman (2015) [31]	64	-3.3	102.5	3253
PLDS 7G	Keigwin & Lehman (2015) [31]	64	-3.3	102.5	3253
PLDS 7G	Keigwin & Lehman (2015) [31]	64	-3.3	102.5	3253
PLDS 7G	Keigwin & Lehman (2015) [31]	64	-3.3	102.5	3253
MV99-GC38	Lindsay et al. (2015) [33]	65	23.2	111.1	1270
MV99-GC38	Lindsay et al. (2015) [33]	65	23.2	111.1	1270
MV99-GC38	Lindsay et al. (2015) [33]	65	23.2	111.1	1270
MV99-MC17/GC32/PC10	Lindsay et al. (2015) [33]	66	23.6	111.6	430
PC08-8-116 cm	Marchitto et al. (2007) [34]	67	23.5	111.6	705
PC08-8-125 cm	Marchitto et al. (2007) [34]	67	23.5	111.6	705
PC08-7-60.5 cm	Marchitto et al. (2007) [34]	68	23.5	111.6	705
PC08-7-80.5 cm	Marchitto et al. (2007) [34]	68	23.5	111.6	705
PC08-7-99.75 cm	Marchitto et al. (2007) [34]	68	23.5	111.6	705
KNR73 6PG	Keigwin & Lehman (2015) [31]	69	4.0	114.2	3805
F2-92-P3	van Geen et al. (1996) [35]	70	35.6	121.6	799
W8709A-13PC	Lund et al. (2011) [36]	71	42.1	123.0	2712
W8709A-13PC	Lund et al. (2011) [36]	71	42.1	123.0	2712
W8709A-13PC	Lund et al. (2011) [36]	71	42.1	123.0	2712
W8709A-13PC	Lund et al. (2011) [36]	71	42.1	123.0	2712
W8709A-13PC	Mix et al. (1999) [37]	71	42.1	125.8	2712
W8709A-13PC	Mix et al. (1999) [37]	71	42.1	125.8	2712
TTN 013-18	Broecker & Clark (2011) [12]	72	-2.0	140.0	4400
ODP 887	Galbraith et al. (2007) [38]	73	54.4	148.5	3647
ODP 887	Galbraith et al. (2007) [38]	73	54.4	148.5	3647
MD02-2489	Rae et al. (2014) [39]	74	55.0	149.0	3640
NES25-1 GGC27	Cook & Keigwin (2015) [40]	75	49.6	150.2	995
B34-91	Cook & Keigwin (2015) [40]	76	49.1	150.3	1227
NES25-1 GGC15	Cook & Keigwin (2015) [40]	77	48.6	150.4	1980
NES25-1 GGC18	Cook & Keigwin (2015) [40]	78	48.8	150.4	1700
NES25-1 GGC20	Cook & Keigwin (2015) [40]	79	48.9	150.4	1510
CHAT-3K	this study	80	-42.7	167.5	4802
CHAT-3K	this study	80	-42.7	167.5	4802
VINO19-4 GGC37	Cook & Keigwin (2015) [40]	81	50.4	167.7	3300
RNDB GGC5	Cook & Keigwin (2015) [40]	82	51.1	167.9	2804
RNDB PC13	Cook & Keigwin (2015) [40]	83	49.7	168.3	2329
RNDB PC13	Cook & Keigwin (2015) [40]	83	49.7	168.3	2329
SO201-2-85KL	Max et al. (2014) [41]	84	57.5	170.4	968

SO201-2-101KL	Cook & Keigwin (2015) [40]	85	58.9	170.6	630
CHAT-5K	this study	86	-40.8	171.5	4240
CHAT-5K	this study	86	-40.8	171.5	4240
RR0503 JPC 36	Sikes et al. (2016) [42]	87	-39.9	176.2	4389
S931	Sikes et al. (2016) [42]	88	-39.5	176.4	4097
RR0503 JPC 79	Sikes et al. (2016) [42]	89	-37.0	176.6	1165
RR0503 TC 83	Sikes et al. (2016) [42]	90	-36.7	176.6	1627
RR0503 TC 83	Sikes et al. (2016) [42]	90	-36.7	176.6	1627
RR0503 TC 83	Sikes et al. (2016) [42]	90	-36.7	176.6	1627
RR0503 TC 83	Sikes et al. (2016) [42]	90	-36.7	176.6	1627
RR0503 JPC 83	Sikes et al. (2016) [42]	91	-36.7	176.6	1627
S794	Sikes et al. (2016) [42]	92	-36.3	176.6	2406
RR0503 JPC 64	Sikes et al. (2016) [42]	93	-37.4	177.0	665
H213	Sikes et al. (2016) [42]	94	-37.0	177.4	2065
SO213-76-2	Ronge et al. (2016) [9]	95	-46.2	178.0	4339
CHAT-16K	this study	96	-42.5	178.5	1408
S938	Sikes et al. (2016) [42]	97	-45.1	179.5	3003
U939	Sikes et al. (2016) [42]	98	-44.5	179.5	1300
CHAT-10K	this study	99	-40.0	180.0	3003
SK129-CR02	this study	100	3.0	-76.0	3800
GIK17940-2	Sarnthein et al. (2015) [43]	101	20.1	-117.4	1727
GIK17940-2	Sarnthein et al. (2015) [43]	101	20.1	-117.4	1727
GIK17940-2	Sarnthein et al. (2015) [43]	101	20.1	-117.4	1727
GIK17940-2	Sarnthein et al. (2015) [43]	101	20.1	-117.4	1727
GIK17940-2	Sarnthein et al. (2015) [43]	101	20.1	-117.4	1727
MD01-2378	Sarnthein et al. (2015) [43]	102	-13.1	-121.8	1738
MD01-2378	Sarnthein et al. (2015) [43]	102	-13.1	-121.8	1738
MD01-2378	Sarnthein et al. (2015) [43]	102	-13.1	-121.8	1738
MD01-2416	Sarnthein et al. (2015) [43]	103	51.3	-167.7	2317
MD01-2416	Sarnthein et al. (2015) [43]	103	51.3	-167.7	2317
PS2644-5	Sarnthein et al. (2015) [43]	104	67.9	21.8	777
PS2644-5	Sarnthein et al. (2015) [43]	104	67.9	21.8	777
PS2644-5	Sarnthein et al. (2015) [43]	104	67.9	21.8	777
PS2644-5	Sarnthein et al. (2015) [43]	104	67.9	21.8	777
PS2644-5	Sarnthein et al. (2015) [43]	104	67.9	21.8	777
PS2644-5	Sarnthein et al. (2015) [43]	104	67.9	21.8	777
PS2644-5	Sarnthein et al. (2015) [43]	104	67.9	21.8	777
PS2644-5	Sarnthein et al. (2015) [43]	104	67.9	21.8	777
KNR 140 JPC22	Keigwin & Swift (2017) [44]	105	28.2	74.4	4712
KNR 140 12JPC	Keigwin & Swift (2017) [44]	106	29.1	54.8	4250
KNR 140 12JPC	Keigwin & Swift (2017) [44]	106	29.1	54.8	4250
KNR197/10 42CDH	Keigwin & Swift (2017) [44]	107	43.5	54.8	3870
KNR197/10 42CDH	Keigwin & Swift (2017) [44]	107	43.5	54.8	3870
KNR197/10 42CDH	Keigwin & Swift (2017) [44]	107	43.5	54.8	3870
KNR197/10 42CDH	Keigwin & Swift (2017) [44]	107	43.5	54.8	3870
KNR140 26GGC	Keigwin & Swift (2017) [44]	108	29.7	73.4	3845
HU89038 PC8	Keigwin & Swift (2017) [44]	109	33.7	57.6	4425

KNR 178 2GGC	Keigwin & Swift (2017) [44]	110	36.1	72.3	3927
KNR 178 2GGC	Keigwin & Swift (2017) [44]	110	36.1	72.3	3927
KNR 178 2GGC	Keigwin & Swift (2017) [44]	110	36.1	72.3	3927
KNR 178 2GGC	Keigwin & Swift (2017) [44]	110	36.1	72.3	3927
KNR197/10 17GGC	Keigwin & Swift (2017) [44]	111	36.4	48.5	5010
PS75/100-4	Ronge et al. (2016) [9]	112	-45.8	-177.1	2498
PS75/100-4	Ronge et al. (2016) [9]	112	-45.8	-177.1	2498
PS75/059-2	Ronge et al. (2016) [9]	113	-54.2	125.4	3613
RR0503 JPC06	Sikes et al. (2016) [42]	114	-45.6	178.8	3385
Sonne 50-37KL	Broecker et al. (1990) [45]	115	18.9	-115.8	2695
Sonne 50-37KL	Broecker et al. (1990) [45]	115	18.9	-115.8	2695
Sonne 50-37KL	Broecker et al. (1990) [45]	115	18.9	-115.8	2695
Vema 28-122	Broecker et al. (1990) [45]	116	11.9	78.7	1800
Vema 28-122	Broecker et al. (1990) [45]	116	11.9	78.7	1800
Vema 28-122	Broecker et al. (1990) [45]	116	11.9	78.7	1800
Knorr 110-82GGC	Broecker et al. (1990) [45]	117	4.3	43.5	2816
Knorr 110-82GGC	Broecker et al. (1990) [45]	117	4.3	43.5	2816
Knorr 110-82GGC	Broecker et al. (1990) [45]	117	4.3	43.5	2816
Knorr 110-66GGC	Broecker et al. (1990) [45]	118	4.6	43.4	3547
Knorr 110-66GGC	Broecker et al. (1990) [45]	118	4.6	43.4	3547
Knorr 110-66GGC	Broecker et al. (1990) [45]	118	4.6	43.4	3547
Knorr 110-50GGC	Broecker et al. (1990) [45]	119	4.9	43.2	3995
Knorr 110-50GGC	Broecker et al. (1990) [45]	119	4.9	43.2	3995
Knorr 110-50GGC	Broecker et al. (1990) [45]	119	4.9	43.2	3995
Knorr 110-50GGC	Broecker et al. (1990) [45]	119	4.9	43.2	3995
ODP 1019	Mix et al. (1999) [37]	120	41.7	124.9	980
ODP 1019	Mix et al. (1999) [37]	120	41.7	124.9	980

Supplementary references

1 Shackleton, N. J. *et al.* Radiocarbon age of last glacial Pacific deep water. *Nature* **335**, 708-711 (1988).

2 Lea, D. W. *et al.* Paleoclimate history of Gala´pagos surface waters over the last 135,000 yr. *Quat. Sci. Rev.* **25**, 1152-1167 (2006).

3 Piotrowski, A. M. *et al.* Indian Ocean circulation and productivity during the last glacial cycle. *Earth Planet. Sci. Lett.* **285**, 179-189 (2009).

4 de la Fuente, M., Skinner, L., Calvo, E., Pelejero, C. & Cacho, I. Increased reservoir ages and poorly ventilated deep waters inferred in the glacial Eastern Equatorial Pacific. *Nat. Commun.* 6:7420 10.1038/ncomms8420 (2015).

5 Waelbroeck, C. *et al.* Distant origin of circulation changes in the Indian Ocean during the last deglaciation. *Earth Planet. Sci. Lett.* **243**, 244-251 (2006).

6 Skinner, L. *et al.* Reduced ventilation and enhanced magnitude of the deep Pacific carbon pool during the last glacial period. *Earth Planet. Sci. Lett.* **411**, 45-52 (2015).

7 McCave, I. N., Carter, L. & Hall, I. R. Glacial - interglacial changes in water mass structure and flow in the SW Pacific Ocean. *Quat. Sci. Rev.* **27**, 1886-1908 (2008).

8 Sadekov, A. Y. *et al.* Palaeoclimate reconstructions reveal a strong link between El Nino-Southern Oscillation and Tropical Pacific mean state. *Nat. Commun.* 4:2692 10.1038/ncomms3692 (2013).

9 Ronge, T. A. *et al.* Radiocarbon constraints on the extent and evolution of the South Pacific glacial carbon pool. *Nat. Commun.* 7:11487 10.1038/ncomms11487 (2016).

10 Keigwin, L. D. & Schlegel, M. A. Ocean ventilation and sedimentation since the glacial maximum at 3 km in the western North Atlantic. *Geochem. Geophys. Geosys.* **3** (2002).

11 Hines, S. K. V., Southon, J. R. & Adkins, J. F. A high-resolution record of Southern Ocean intermediate water radiocarbon over the past 30,000 years. *Earth Planet. Sci. Lett.* **432**, 46-58 (2015).

12 Broecker, W. & Clark, E. Radiocarbon-age differences among coexisting planktic foraminifera shells: The Barker Effect. *Paleoceanography* **26** (2011).

13 Ahagon, N., Ohkushi, K., Uchida, M. & Mishima, T. Mid-depth circulation in the northwest Pacific during the last deglaciation: Evidence from foraminferal radiocarbon ages. *Geophys. Res. Lett.* **30**, 2.1-2.4 (2003).

14 Okazaki, Y., Sagawa, T., Asahi, H., Horikawa, K. & Onodera, J. Ventilation changes in the western North Pacific since the last glacial period. *Clim. Past* **8** (2012).

15 Murayama, M., Taira, A., Iwakura, H., Matsumoto, E. & Naka- mura, T. Northwest Pacific deep water ventilation rate during the past 35,000 years with the AMS 14C foraminifera ages. *Summaries of Researches Using AMS at Nagoya University (Nagoya University Center for Chronological Research, Nagoya, Japan)* **3**, 114–121 (1992).

16 Broecker, W., Clark, E. & Barker, S. Near constancy of the Pacific Ocean surface to mid-depth radiocarbon-age difference over the last 20 kyr. *Earth Planet. Sci. Lett.* **274**, 322-326 (2008).

17 Broecker, W. *et al.* Ventilation of Glacial Deep Pacific Ocean. *Science* **306**, 1169-1172 (2004).

18 Bryan, S. P., Marchitto, T. M. & Lehman, S. J. The release of ^{14}C-depleted carbon from the deep ocean during the last deglaciation: Evidence from the Arabian Sea. *Earth Planet. Sci. Lett.* **298**, 244-254 (2010).

19 Barker, S., Knorr, G., Vautravers, M., Diz, P. & Skinner, L. C. Extreme deepening of the Atlantic overturning circulation during deglaciation. *Nature Geoscience* **3** (2010).

20 Thornalley, D. J. R. *et al.* A warm and poorly ventilated deep Arctic Mediterranean during the last glacial period. *Science* **349**, 706-710 (2015).

21 Freeman, E., Skinner, L., Waelbroeck, C. & Hodell, D. Radiocarbon evidence for enhanced respired carbon storage in the deep Atlantic at the Last Glacial Maximum. *Nat. Commun.* 7:11998 10.1038/ncomms11998 (2016).

22 Skinner, L., Waelbroeck, C., Scrivner, A. & Fallon, S. Radiocarbon evidence for alternating northern and southern sources of ventilation of the deep Atlantic carbon pool during the last deglaciation. *Proceedings of the National Academy of Sciences* **111**, 5480–5484 (2014).

23 Cleroux, C., Demenocal, P. & Guilderson, T. Deglacial radiocarbon history of tropical Atlantic thermocline waters: absence of CO_2 reservoir purging signal. *Quat. Sci. Rev.* **30**, 1875-1882 (2011).
24 Skinner, L. C., Fallon, S., Waelbroeck, C., Michel, E. & Barker, S. Ventilation of the deep Southern Ocean and deglacial CO_2 rise. *Science* **328**, 1147-1151 (2010).
25 Thornalley, D. J. R., Barker, S., Broecker, W., Elderfield, H. & McCave, I. N. The deglacial evolution of North Atlantic deep convection. *Science* **331**, 202-205 (2011).
26 Chen, T. *et al.* Synchronous centennial abrupt events in the ocean and atmosphere during the last deglaciation. *Science* **349**, 1537-1541 (2015).
27 Sortor, R. N. & Lund, D. C. No evidence for a deglacial intermediate water Delta C-14 anomaly in the SW Atlantic. *Earth Planet. Sci. Lett.* **310**, 65-72 (2011).
28 Burke, A. & Robinson, L. F. The Southern Ocean's role in carbon exchange during the last deglaciation. *Science* **335**, 557-561 (2012).
29 De Pol-Holz, R., Keigwin, L. D., Southon, J., Hebbeln, D. & Mohtadi, M. No signature of abyssal carbon in intermediate waters off Chile during deglaciation. *Nature Geoscience* **3**, 192-195 (2010).
30 Siani, G. *et al.* Carbon isotope records reveal precise timing timing of enhanced Southern Ocean upwelling during the last deglaciation. *Nat. Commun. 7*:2758 DOI:10.1038/ncomms3758 (2013).
31 Keigwin, L. D. & Lehman, S. J. Radiocarbon evidence for a possible abyssal front near 3.1 km in the glacial equatorial Pacific Ocean. *Earth Planet. Sci. Lett.* **425** (2015).
32 Stott, L., Southon, J., Timmermann, A. & Koutavas, A. Radiocarbon age anomaly at intermediate water depth in the Pacific Ocean during the last deglaciation. *Paleoceanography* **24**, PA2223 (2009).
33 Lindsay, C. M., Lehman, S. J., Marchitto, T. M. & Ortiz, J. D. The surface expression of radiocarbon anomalies near Baja California during deglaciation. *Earth Planet. Sci. Lett.* **422**, 67-74 (2015).
34 Marchitto, T. M., Lehman, S. J., Otiz, J. D., Fluckiger, J. & van Geen, A. Marine radiocarbon evidence for the mechanism of deglacial atmospheric CO_2 rise. *Science* **316**, 1456-1459 (2007).
35 van Geen, A. *et al.* Ventilation changes in the northeast Pacific during the Last Deglaciation. *Paleoceanography* **11**, 519-528 (1996).
36 Lund, D. C., Mix, A. C. & Southon, J. Increased ventilation age of the deep northeast Pacific Ocean during the last deglaciation. *Nature Geoscience* **4**, 771-774 (2011).
37 Mix, A. C. *et al.* in *Mechanisms of global climate change at millennial time scales* Vol. 112 (eds P.U. Clark, R.S. Webb, & L.D. Keigwin) 127-148 (American Geophysical Union, 1999).
38 Galbraith, E. *et al.* Carbon dioxide release from the North Pacific abyss during the last deglaciation. *Nature* **449**, 890-893 (2007).
39 Rae, J. W. B. *et al.* Deep water formation in the North Pacific and deglacial CO2 rise. *Paleoceanography* **29**, 645-667 (2014).

40 Cook, M. & Keigwin, L. D. Radiocarbon profiles of the NW Pacific from the LGM and deglaciation: Evaluating ventilation metrics and the effect of uncertain surface reservoir ages. *Paleoceanography* **30**, 174-195 (2015).

41 Max, L. *et al.* Pulses of enhanced North Pacific Intermediate Water ventilation from the Okhotsk Sea and Bering Sea during the last deglaciation. *Clim. Past* **10**, 591-605 (2014).

42 Sikes, E. L., Cook, M. S. & Guilderson, T. P. Reduced deep ocean ventilation in the Southern Pacific Ocean during the last glaciation persisted into the deglaciation. *Earth Planet. Sci. Lett.* **438**, 130-138 (2016).

43 Sarnthein, M., Balmer, S., Grootes, P. & Mudelsee, M. Planktic and Benthic ^{14}C Reservoir Ages for Three Ocean Basins, Calibrated by a Suite of ^{14}C Plateaus in the Glacial-to-Deglacial Suigetsu Atmospheric ^{14}C Record. *Radiocarbon* **57**, 129-151 (2015).

44 Keigwin, L. D. & Swift, S. A. Carbon isotope evidence for a northern source of deep water in the glacial western North Atlantic. *Proceedings of the National Academy of Sciences* (2017).

45 Broecker, W. S., Peng, T. H., Trumbore, S., Bonani, G. & Wolfli, W. The distribution of radiocarbon in the glacial ocean. *Global Biogeochemical Cycles* **4**, 103-117 (1990).
